# Supplementary material for: Dynalign II: common secondary structure prediction for RNA homologs with domain insertions
Source: Nucleic Acids Res. 2014 Nov 21;42(22):13939–48. doi: 10.1093/nar/gku1172 (PMC4267632; doi:10.1093/nar/gku1172)
Supplement: SUPPLEMENTARY DATA [file supp_gku1172_nar-02021-z-2014-File012.zip › manual/Overview/index.html]

RNAstructure Installation and Overview


|  |  |  |
| --- | --- | --- |
|  | RNAstructure Installation and Overview  Version 5.7 | - Contents - Index |
| RNAstructure is a product of the Mathews Lab, University of Rochester Medical Center, Department of Biochemistry and Biophysics.  **Continued development of RNAstructure is made possible by the support of NIH grant R01GM076485.**  How to Install the Pre-Built Packages:  Installation Instructions for Linux Systems  Step-by-step instructions detailing how to install theRNAstructure package on Linux systems.  Installation Instructions for Macintosh Systems  Step-by-step instructions detailing how to install the RNAstructure package on Macintosh systems.  Installation Instructions for Windows Systems  Step-by-step instructions detailing how to install the RNAstructure package on Windows systems.  Other Help Instructions:  Building Instructions  Guide to building the programs in the RNAstructure repository.  Building Requirements  Guide to external programs or variables necessary to build the RNAstructure repository tools.  Cleaning the Repository  Important information about how the RNAstructure repository conducts cleaning operations.  Components  A complete list of components of the RNAstructure software package, including references to the literature.  Contributors  A list of people who contributed to the code base of RNAstructure.  Interfaces Summary  A detailed summary of each interface in the RNAstructure repository, including name, location, and building commands.  Libraries  Descriptions of the libraries that are built by the RNAstructure repository.  Makefiles  Detailed descriptions about how each of the Makefiles in the RNAstructure repository work.  Object Files  Important information about object file creation when building the text interfaces and Java GUI in the RNAstructure repository.  Repository Organization  Details on how the RNAstructure repository is organized.  Running the RNAstructure JAVA GUI  Special considerations to be aware of when attempting to run the JAVA GUI.  Update History  History of changes and updates to the RNAstructure repository. | | |
| Visit The Mathews Lab RNAstructure Page for updates and latest information. | | |
